# Ultrasound-guided Erector Spinae Plane Block for the Management of Herpes Zoster Pain: Observational Study

**DOI:** 10.7759/cureus.5891

**Published:** 2019-10-11

**Authors:** Tayfun Aydın, Onur Balaban, Ali Ahiskalioglu, Haci A Alici, Aysenur Acar

**Affiliations:** 1 Anesthesiology & Pain Medicine, Kutahya Health Sciences University, Kutahya, TUR; 2 Anesthesiology, Ataturk University School of Medicine, Erzurum, TUR; 3 Pain Medicine, Ataturk University School of Medicine, Erzurum, TUR; 4 Anesthesiology & Reanimation, Kutahya Health Sciences University, Kutahya, TUR

**Keywords:** herpes zoster, erector spinae plane block, ultrasound, in-plane technique, acute herpetic pain, single shot injection, continuous analgesia, post-herpetic neuralgia

## Abstract

Background

Herpes zoster is caused by the reactivation of latent varicella-zoster virus, which promotes acute and chronic pain that may interfere with daily activities and reduce the quality of life. Ultrasound-guided erector spinae plane (ESP) blocks are used for a wide variety of indications in the management of acute, chronic, and postoperative pain. Our aim was to evaluate the efficacy of ultrasound-guided erector spinae plane blocks for the management of pain in herpes zoster.

Methods

The medical records of 34 patients with acute or chronic pain during herpes zoster between May 2017 and June 2018 were investigated at two pain clinic centers. The patients received ultrasound-guided erector spinae plane block: We performed a single injection for the patients having acute pain and a continuous block for the patients having chronic pain. Patient characteristics, block characteristics (needle insertion level, catheter, or single insertion), the volume of given local anesthetics, the intensity of pain before and after the block procedure using a numerical rating score (NRS) between 0 and 10, and the duration of analgesia were evaluated.

Results

All patients reported a remarkable and rapid resolution of pain immediately after the block procedure. Median (min-max) NRS score before the block procedure was 9 (4-10). The median (min-max) NRS score was 1.5 (0-7) after the block procedure. The difference was found to be statistically significant (p<0.0001). NRS score after the third month was 1 (0-3); the difference is statistically significant (p=0.002). The median value of analgesia time (min-max) was 18 (3-24) hours.

Conclusion

Our preliminary experience demonstrated that an ESP block provided sufficient analgesia in acute herpetic pain. A combination of ESP block, pregabalin, and tramadol was also effective within the three-months-period after the block performance.

## Introduction

Herpes zoster is caused by the reactivation of latent varicella-zoster virus. Acute severe pain and post-herpetic neuralgia (PHN) is a feared complication of herpes zoster infection. The reported incidence of PHN varies between 5% and 50% depending on the study design, age distribution, and definition [1-3]. Treatment of pain in the acute phase of herpes zoster has great importance due to the possible development of PHN. The pain may be severe, persist for months or years, interfere with sleep, and affect the quality of life. Acute and chronic post-herpetic pain is challenging to manage which may require interventional regional analgesia methods in addition to conventional medical therapy [1,4-5].

Ultrasound (US)-guided erector spinae plane (ESP) block is one of the interfascial plane blocks that target the dorsal and ventral rami of the spinal nerves [6]. Although there is not sufficient evidence for the spread of local anesthetic to the ventral rami, recent anecdotal reports demonstrated effective postoperative analgesia after thoracic and lumbar surgeries affecting both the ventral and dorsal rami [7-9]. ESP blocks were also effective for pain relief in chronic thoracic pain, rib fractures, and pulmonary malignancy [10-12]. The ESP block has a clear and simple sonoanatomy, is easy to perform, and is well-tolerated by patients [7]. The single-shot injection technique or continuous analgesia with a catheter placement is possible. The block could be performed unilaterally or bilaterally, depending on the requirement of analgesia [7,11]. ESP blocks could potentially provide effective analgesia and might be a successful method in the treatment of the acute and chronic pain associated with herpes zoster. Our aim was to demonstrate the efficacy of the ESP block for the management of pain in acute herpes zoster and post-herpetic neuralgia. We hypothesized that pain might be significantly less than before, after the performance of ESP blocks in patients with herpetic pain.

## Materials and methods

**Figure 1:**
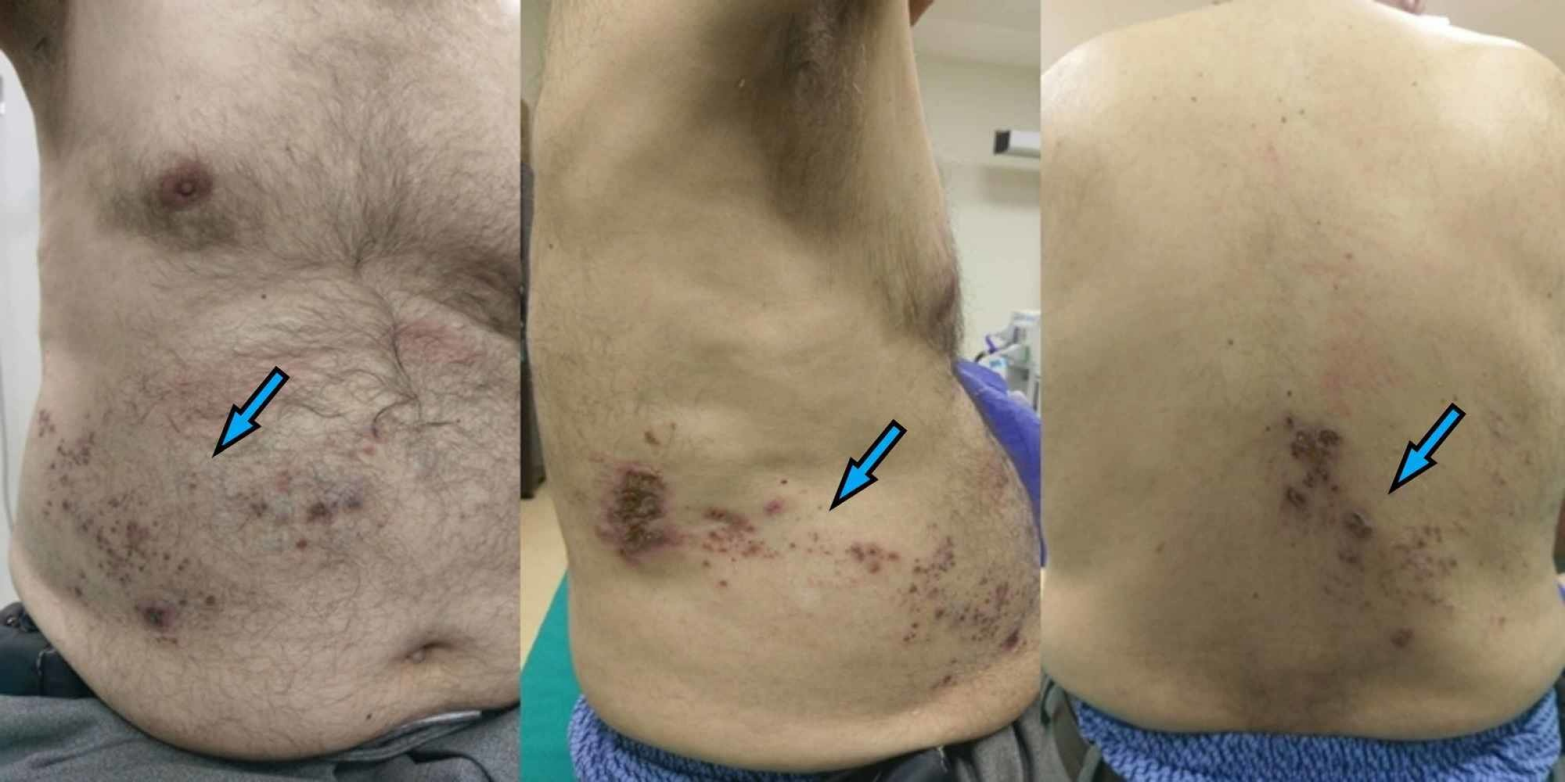
Unilateral herpetic rash in a patient at the lower thoracic dermatomal segments Blue arrows indicate herpetic rash

This research was designed as a multicenter retrospective observational study. Local institutional review board (IRB) approval was provided (IRB no:2018/31) and written informed consent was obtained from the patients for the performance of erector spinae plane blocks and for the publication of this report. The medical records of patients having herpes zoster (Figure 1) who underwent erector spinae plane block in two territory hospitals for the management of acute or chronic pain, between May 2017 and June 2018, were investigated. Patient characteristics (age, body mass index (BMI), dermatomal level of vesicles, and previous medication), block characteristics (needle insertion level, catheter, or single insertion), volume of local anesthetics, intensity of pain before and after the block procedure using a numerical rating score between 0 and 10 (NRS), and duration of analgesia were evaluated. Duration of analgesia was defined as the analgesia starting from the performance of the block until the first analgesic requirement or reporting a pain score of 4/10. Patients with insufficient follow-up or with missing data were excluded from the study.

Block method

**Figure 2:**
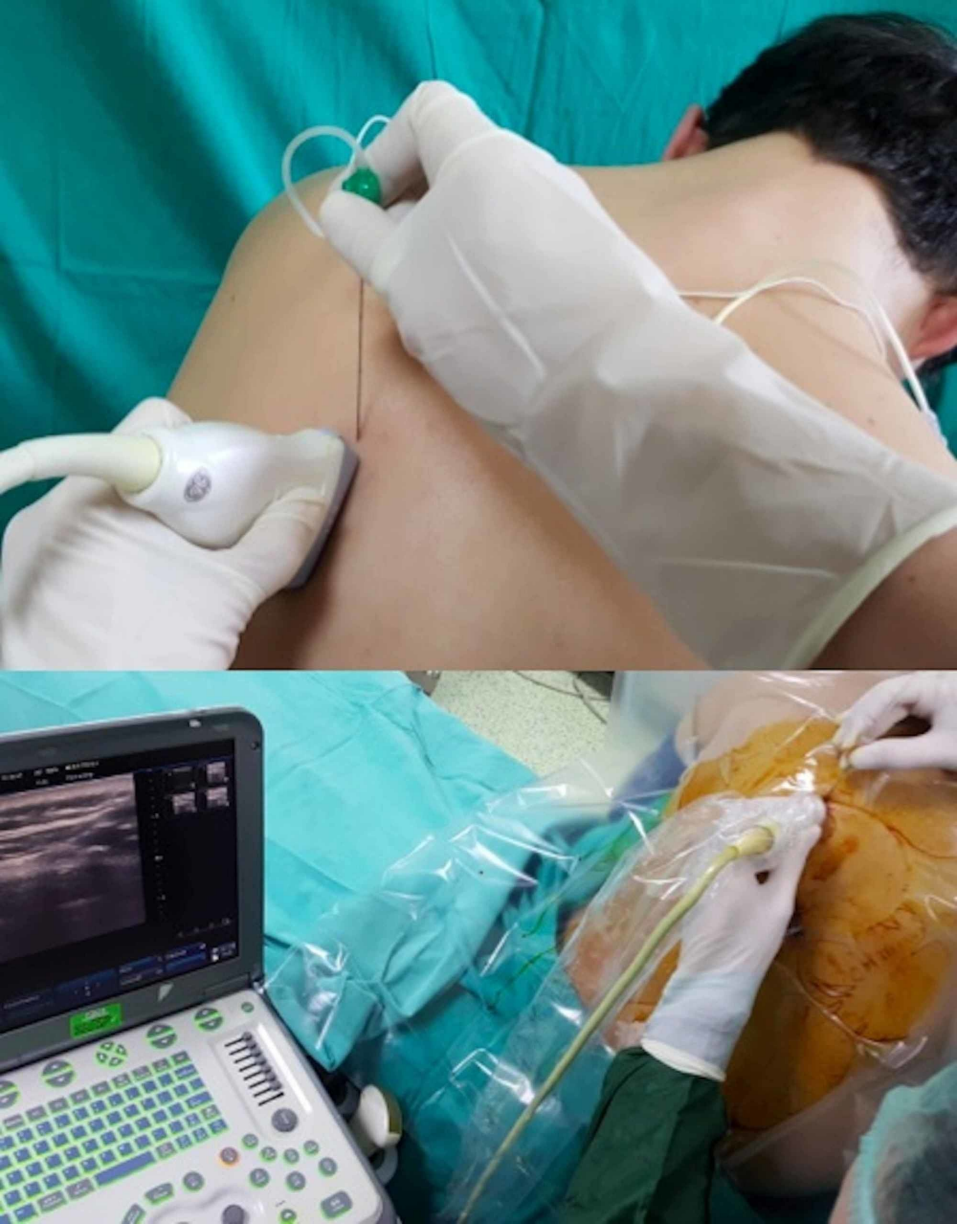
The position of the patient, ultrasound probe, and needle during the performance of an erector spinae plane block The ultrasound probe is placed 2-3 cm lateral to the spinous processes in the longitudinal plane. The needle is inserted from the cephalad aspect of the probe and advanced in the caudal direction within the plane of the ultrasound beam.

**Figure 3:**
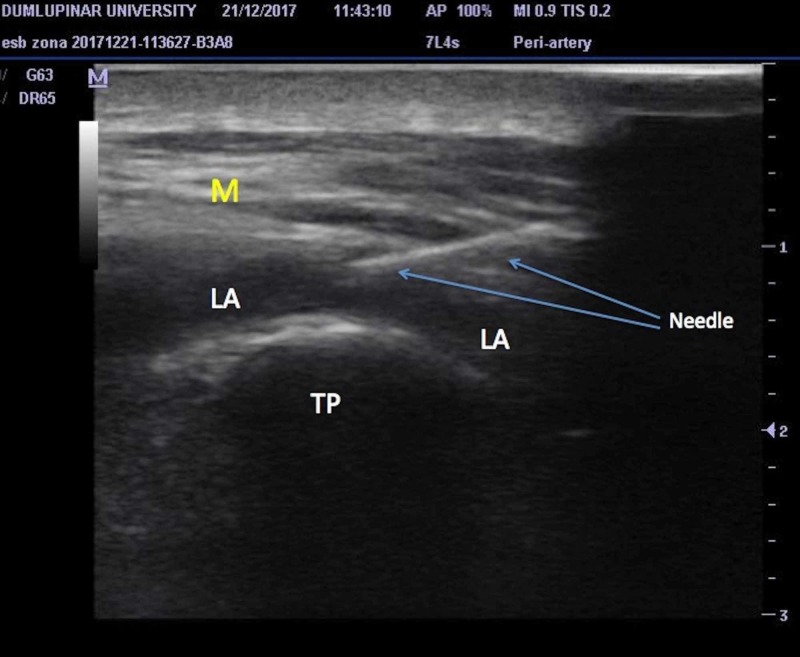
Ultrasound image of the needle Needle, transverse process of vertebra (TP), spinal muscles (M) and spread of local anesthetic solution (LA) during the performance of erector spinae plane block.

A unilateral erector spinae plane block was applied to all patients on the affected side (Figure 1). An 8 cm echogenic needle (Stimuplex® Ultra 360® Braun, Melsungen, Germany) was used for the performance of a single injection block and a 20-G epidural catheter through an 80-mm-18-G Tuohy needle (Epifix, Egemen® International, Izmir, Turkey) was placed for continuous blocks. A single injection was performed for patients having acute pain and a catheter was placed for continuous analgesia in patients having chronic pain. During the block procedure, the patients were placed in the sitting position with their arms resting on a pillow (Figure 2). The anesthetist performed the procedure in the sitting position at the dorsal side of the patient with the US machine in front of the performer. We counted the laminae in the caudad-to-cephalad direction, starting from the sacrum using the US to determine the exact vertebral level where the needle would be inserted. A low-frequency curved array US probe was used to determine the targeted transverse process and for the performance of blocks at the lumbar level. A linear high-frequency ultrasound probe is used to perform the blocks at the thoracic level. After the determination of the needle insertion site, the US probe was placed over the spinous processes of the vertebrae at the midline in the longitudinal plane. The US probe was then slid laterally from the spinous processes to visualize the transverse process (Figure 2). The transverse process was visualized in the US images as a thin, 0.5-1 cm long hyperechoic line with a hypoechoic bony shadow underneath (Figure 3). Over the transverse process, paraspinal muscles (erector spinae muscle, trapezius, rhomboid major, or latissimus dorsi muscles depending on the vertebral level) were identified. The spinal muscles are usually visualized as multiple hyperechoic lines (representing fascia and muscle fibers) with hypoechoic areas between them (Figure 3). In US images, the erector spinae muscle is seen adjacent to the transverse processes (Figure 3).

**Figure 4:**
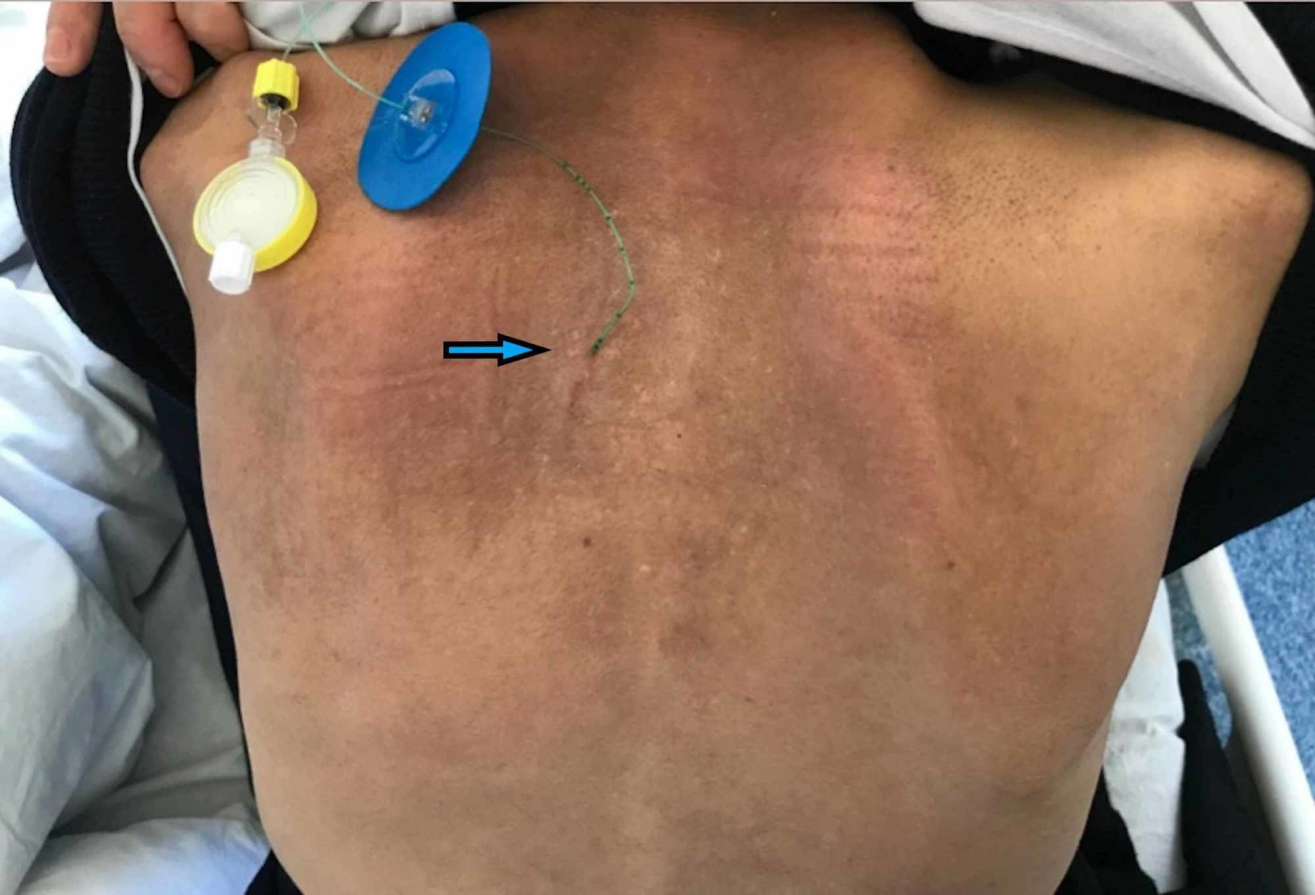
Catheter placed in the erector spinae plane for continuous unilateral analgesia. The catheter is placed longitudinally, 2,5 cm lateral to the spinous processes. Blue arrows indicate catheter localization.

When the transverse process was identified, the probe was kept still and the needle was inserted from the cephalad aspect of the US probe (Figure 2). The needle was advanced posterior-to-anteriorly, in the cephalad to caudal direction using the in-plane orientation within paraspinal muscles, targeting the transverse process. When the tip of the needle reached and contacted the transverse process, the local anesthetic drugs were injected, aiming to distribute within the plane between the anterior fascia of the erector spinae muscle and the transverse process. The needle tip and the spread of the local anesthetic solution were visualized in real time using the plane technique (Figure 3). Twenty ml of local anesthetic (bupivacaine, 0.25% concentration) was administered as standard in all patients based on the published studies. In case of a continuous block, a 20-gauge epidural catheter was placed through a Tuohy needle in the plane that was enlarged by injected local anesthetic volume (Figure 4). The catheter was secured in place using the fixing apparatus coming out from the catheter set.

The frequency of injections and catheter top-ups were as follows: A single injection ESP block was performed in patients with new-onset pain. One injection was administered and the patient was discharged the same day. Afterward, the patient was followed up by telephonic communication. The intensity of pain was evaluated by asking the patients to give a score between 0 and 10 using the NRS. Pregabalin treatment was started and continued for three months. No additional injection was performed within the three months period. A continuous block was applied by placing a catheter for patients having chronic pain. The patients were followed for three months by telephonic communication. Pregabalin treatment was also started. Ten ml of 0.25% bupivacaine was administered from the catheter every 12 hours depending on the analgesic requirement of the patients. The catheters were kept in place as long as the patients required catheter top-ups when the patients did not require more local analgesics, the catheter was then removed. The patient was discharged the same day and the catheter top-ups were performed by the patient or a caregiver at home after a short training. The catheters were kept in place for a maximum time of 3 months. Tramadol was provided in case of a need for rescue analgesia. 

Data were analyzed using the SPSS 21 (SPSS Inc., Chicago, IL, USA) package program. Continuous quantitative data were presented as number, mean ± standard deviation. The paired sample test was applied to non-parametric data for the statistical evaluation of repeated measurements. P<0.05 was considered significant.

## Results

Forty-two patients met the inclusion criteria and eight patients were excluded due to insufficient data. Fourteen patients were male and 20 patients were female. An ESP catheter was placed in 11 patients and a single injection ESP block was performed in 23 patients. Patient characteristics are summarized in Table 1.

Needle insertion levels were at T4 in eight patients, T7 in four patients, T9 in four patients, T6 in three patients, T5 in three patients, T10 in three patients, T12 in three patients, T8 in two patients, T1 and T2 in one patient. There was one lumbar puncture at the L2 level and one bi-level injection at T4-T5 levels. The terminal location of catheter tips was at T4 level in three patients, T5 level in two patients, T10 level in two patients, and the T4, T6, T7, and T9 levels in one patient.

**Table 1:** Summary of patient demographic and pain characteristics The values are presented as mean ± standard deviation, median (minimum-maximum) or number of patients. Low thoracic indicates T8 and below injection points.

Demographic and pain characteristics (n=34)	
Age (year)	63.62 ± 12.06
Male/female (n)	14/20
Weight (kg)	77.09 ± 19.43
Height (cm)	164.15 ± 7.39
BMI (kg/m2)	28.46 ± 6.07
Onset of disease (day)	33.74 ± 44.92
Duration of analgesia (hours)	18 (3-24)
Injection (continuous/single)	11/23
Needle insertion level (thoracic/low thoracic)	21/13
Dermatomal level of vesicles (thoracic/thoracolumbar)	27/7

All blocks were uneventful and no remarkable complication occurred during and after the performance of the blocks. No clinically apparent motor blockade was observed in any of the patients. No complications were observed regarding long-term catheterization such as signs of infection.

**Figure 5:**
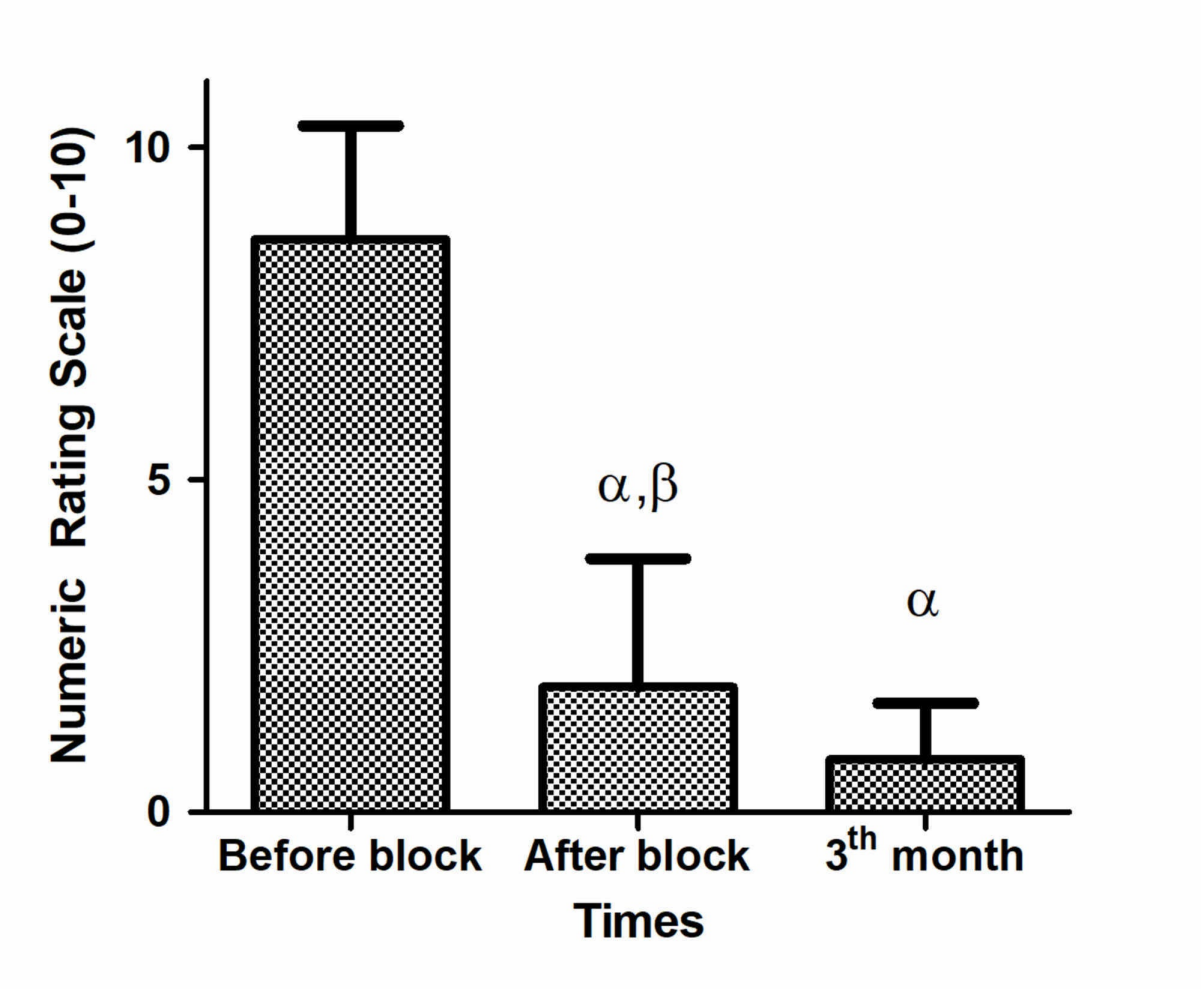
Numeric Rating Scale scores (0-10) ^α^ Between before vs after block and third month, p<0.0001 paired samples test ^β^ Between after vs third month, p=0.002 paired samples test

The median value of analgesia time (min-max) was 18 (3-24) hours. The median (min-max) NRS score before the block procedure was 9 (4-10). The median (min-max) NRS score was 1.5 (0-7) after the block procedure. The difference was found to be statistically significant (p<0.0001). The median (min-max) NRS score after the third month was 1 (0-3); the difference is statistically significant (Table 2 and Figure 5).

**Table 2:** Summary of pain Numeric Rating Scale scores (0-10) and patient satisfaction The values are presented as median (minimum-maximum) or number. ^α^ Between before vs after block and third month, p<0.0001 paired samples test ^β^ Between after vs third month, p=0.002 paired samples test

Pain intensity before block (0-10)		9 (4-10)
Pain intensity after block (0-10)		1.5 (0-7)^ α, ^^β^
Pain intensity after third month (0-10)		1 (0-3)^ α^
Patient satisfaction	Poor	0
	Fair	1
	Good	12
	Excellent	21

All patients reported a remarkable and rapid resolution of pain immediately after the block procedure.

## Discussion

The current study demonstrates that an ESP block provides immediate acute pain relief in patients with severe pain due to herpes zoster infection. Also, the ESP block provided sufficient long-term analgesia in these patients when used in combination with oral pregabalin and tramadol. The ESP block was successful at various dermatomal levels in the thoracic and lumbar regions.

Severe acute pain is one of the factors that was found strongly associated with an increased risk of post-herpetic neuralgia [13]. Early intervention is important for resolving zoster pain. Even with adequate medical treatment using antiepileptics, analgesics, and antivirals, some patients do not have sufficient pain relief and may need additional interventional procedures. Various nerve blocks are found to provide effective analgesia and prevent progression to post-herpetic neuralgia by decreasing the painful stimuli and alleviating central sensitization during the acute phase of herpes zoster infection [3].

Sympathetic and somatic block techniques for treating herpetic pain include central neural blocks (mostly continuous epidural block), paravertebral blocks, stellate ganglion blocks, intercostal blocks, cervical plexus blocks, and intra-cutaneous injections. Intrathecal steroid and local anesthetic injections, various peripheral nerve blocks (such as occipital, supraorbital, supratrochlear, and infraorbital) have also been used [2]. Up to date, there is no study supporting the efficacy of the ESP block in herpes zoster pain. The implantation of spinal cord stimulators (SCS) was offered, however, there is no randomized controlled study that supports the efficacy of SCS [14]. The preventive effects of an early nerve block may be potent when performed with continuous treatment modalities than single administration [2]. The effectiveness of pulsed radiofrequency to the dorsal root ganglion has been reported as well, in treating pain after the acute phase of zoster [4].

The ESP block was first described by Forero et al. for the management of thoracic pain with an injection at the thoracic vertebra level [6,10]. The block is thought to work with the diffusion of local anesthetic into the paravertebral and intercostal spaces. The observed analgesic effect suggests that it is able to block the thoracic spinal nerves. Anatomical and radiological investigations indicate that the site of action is at the dorsal and ventral rami of the spinal nerves [6,15]. The diffusion of local anesthetic was observed in the paravertebral and intercostal spaces [16-17]. Studies have shown that the spread of local anesthetic in the paravertebral space in the cephalic and caudal direction can lead to analgesia from C7-T2 to L4-5, depending on the injection level [16,9]. In the cases of Forero et al., the administration of 25 mL local anesthetic provided significant analgesia three dermatomes cephalad and five dermatomes caudad from the injection site [10]. One previous investigation on fresh cadavers demonstrated that 20 mL injected at the level of T5 spreads from the T2 to T8 vertebral levels [6]. The distribution of local anesthetics and the analgesic characteristics of the block may be affected by the injection point and the volume and concentration of local anesthetics. Analgesia was provided, usually one-to-two dermatome cephalad and caudad levels from where the solution was injected, using 10 mL local anesthetic in our cases.

ESP blocks were successfully performed in various cases having chronic pain [10,12,16-19]. There is one individual case in which an ESP block was successfully used for the management of acute herpes zoster pain but its effect in chronic neuropathic pain is still unclear [19]. Up to date, there is insufficient data and a need for long-term studies to assess the effect of ESP blocks for preventing post-herpetic neuralgia.

One advantage of this technique is the achievement of extensive analgesia with a single shot injection. A single injection ESP block provided analgesia for up to 24 hours in our cases. It was shown to provide long-lasting postoperative analgesia up to 48 hours [20]. Nevertheless, we suggest placing an ESP catheter for the chronic pain associated with herpes zoster. Long-term catheterization maybe sometimes problematic and intolerable, but it provides very effective and prolonged pain relief. Our patients were able to administer local anesthetic drugs from the catheter without the help of a pain nurse after a short training and most of the treatment continued at home.

Another advantage of an ESP block is the lower potential risk of mechanical complications, such as nerve damage, pleural puncture, or vessel puncture than its alternatives: paravertebral or intercostal nerve blocks. An ESP block is technically easier to perform without the necessity of multiple injections performed in the intercostal nerve block. ESP blocks have also the advantage of either unilateral or bilateral application. We performed unilateral blocks in patients with herpes zoster requiring unilateral analgesia. The rash of herpes zoster is dermatomal and does not cross the midline, consistent with reactivation from the dorsal root or cranial nerve ganglia. Thus, the possibility of a unilateral blockade should make ESP blocks of choice in Herpes zoster pain. The procedure could be performed at the patient’s bedside or in a block room, either in the sitting or the prone position. Simple monitoring (pulse oximetry, electrocardiogram, and noninvasive blood pressure monitoring) should be applied, and an intravenous line should be placed for the safety of the procedure.

The main limitation of this study is that its retrospective and non-blinded nature may limit us to compare this novel technique to other interventional analgesia methods. Another limitation is that the analgesic efficacy of the ESP block in the three-month-period may be due to the effect of pregabalin and tramadol treatment. On the other hand, we cannot usually conduct a pain study with a control group that no medication or placebo is given due to ethical reasons. Thus, we could not be able to design a study with a control or placebo group to evaluate the efficacy of the ESP block alone. The long-term efficacy of local anesthetics may be related to their desensitization effect but making that differentiation was not possible in our study design.

## Conclusions

In conclusion, our preliminary experience demonstrated the efficacy of ESP blocks for the management of acute herpetic pain. A combination of ESP block and oral pregabalin with oral tramadol also provided sufficient analgesia in the three-month-period after block performance. An ESP block is easy to apply under ultrasound guidance with an easy sonoanatomy to understand. Adequate analgesia was achieved with the application of a single-injection block or catheter placement in our study. The ESP block may be a promising method for pain management in herpes zoster as an alternative to thoracic paravertebral blocks and intercostal blocks. Comparative studies are needed to assess the superiority of the ESP block to other interventional regional analgesia methods.
